# The community residents’ NIMBY attitude on the construction of community ageing care service centres: a cross-sectional study

**DOI:** 10.1186/s12913-022-07478-5

**Published:** 2022-01-21

**Authors:** Bo Yu, Yue-Hong Han, Yin Sun, Xu-Dong Zhang

**Affiliations:** 1 Faculty of Management and Economics, Kunming University of Science and Technology, Kunming, China; 2 School of Humanities and Management, Yunnan University of Chinese Medicine, Kunming, China; 3 School of Marxism, Kunming University of Science and Technology, Kunming, China; 4 School of Marxism, Yunnan University of Business Management, Kunming, China

**Keywords:** Community Aged Care Service Centre (CACSC), NIMBY attitude, Superstitious belief

## Abstract

**Background:**

China is the country with the largest elderly population. To actively respond to this ageing population, China has proposed the Community Aged Care Service Centre (CACSC) network as the major elderly care development policy. However, many residents resisted the development of the CACSC network, which affected its smooth implementation. Based on the theory of “Not in My Backyard” (NIMBY), this paper proposes a model of the influencing factors of community residents on the opposition to the construction of CACSCs.

**Methods:**

Residents living in urban communities over the age of 20 in China are the target of this study. The questionnaires were collected in the form of electronic questionnaires created on a professional website, and hyperlinks to the questionnaires were distributed through social media. The descriptive statistical analysis, T-tests, ANOVA and structural equation modelling were performed on cross-sectional survey data from 509 questionnaires.

**Results:**

The research results show that superstition, the NIMBY attitude, and perceived risk have a significant positive impact on the opposition to the construction of CACSCs, while the negative impact of perceived benefit on the opposition intention is not significant. Moreover, perceived knowledge has a significant positive impact on perceived benefit and a significant negative impact on superstition and perceived risk.

**Conclusions:**

Strengthen policy advocacy on ageing, clarify the service content of CACSC and encourage young people to participate in public welfare activities for the elderly can reduce the opposition of community residents to the construction of a CACSC.

## Background

Today, China is the country with the largest number of elderly people. According to the results of the seventh national census in 2020, 264 million people over 60 years old resided in the country, accounting for 18.7% [1]. The characteristics of getting older before getting rich, rapidly ageing, and a large-scale elderly population will be important national conditions for an extended period in China. In October 2020, China upgraded “actively responding to population ageing” as a national strategy, which proposed vigorously developing inclusive elderly care services, including building an elderly care service system that coordinated home and community institutions, combining medical care and improving community home care. In addition, the service system would also promote the transformation of public facilities to adapt to ageing, expand the supply of beds in elderly care institutions, and improve their service capabilities and levels [2].

According to the experience of countries that have entered an ageing society earlier, due to the poor reputation of residential aged care facilities (RACFs), the thought of spending their final years in an RACF often makes many elderly people feel terrified [3]. Therefore, an increasing number of elderly people choose to live in their own homes and communities. Governments have also increased their support and funding for the Community Aged Care Service Centre (CACSC) network [4]. For example, Sweden is one of the first countries to propose an open care model for the elderly and clearly phase out nursing homes [5]. Maddocks [3] concluded a new home care model with international application potential, that is, building a community rehabilitation centre that provides support for home care in communities. Moreover, Frochen et al. [6] conducted a study of the spatial distribution of community elderly care facilities in the Los Angeles area of the United States and found that areas with small- and medium-sized facilities are more likely to provide the nearby population with the largest capacity. The CACSC network in this study provides a combination of medical care and nursing care. The CACSC network provides not only services such as living, group meals, life care, rehabilitation care, spiritual comfort, and cultural entertainment but also long-term care for bedridden elderly patients, disabled patients, and terminally ill patients who need tranquillity care.

Home care is the first choice for many elderly people. A survey shows that only 4.38% of the elderly in China are willing to live in institutions, and 95.6% of the elderly want to spend their final years at home [7]. However, limited by the health status of the elderly, family structure, inconvenience of living and other issues, traditional family care cannot meet the needs of home care. Therefore, the CACSC network emerged. Currently, various cities in China are actively promoting the construction of CACSCs. According to statistics from the Ministry of Civil Affairs, as of August 2020, more than 180,000 CACSCs exist nationwide, accounting for 83.6% of the total number of elderly care institutions and facilities.

However, some problems were also encountered. Since 2014, the media has shown that community owners from all over China, such as Hangzhou, Nanjing, Changsha, Jinan, Zhuhai, and Kunming, have resisted the construction of CACSCs. The proprietors mainly expressed their opposition by hanging banners, reporting to the government, and exposing them to the media. In particular, these proprietors strongly resisted the CACSC network with medical and nursing functions, providing tranquillity care and other services.

**Fig. 1:**
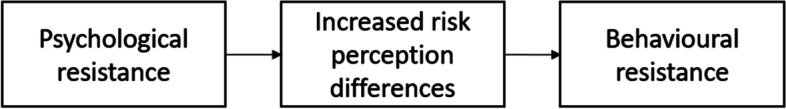
Conflict reaction chain

Xu and Jing [8] conducted a qualitative analysis of the conflicts in the establishment of Wuhan rehabilitation hospitals in 2015 and discovered that the core of the escalation and evolution of community elderly care facilities is a process of a conflict reaction chain. From psychological resistance, it develops into increased risk perception and evolves into behavioural resistance as Fig. 1 shown. Moreover, Tang [9] selected a community in Changsha as an example to analyse the “Not in My Backyard (NIMBY)” attitude of elderly care institutions and pointed out that elderly care institutions are new types of neighbouring avoidance facilities. Due to their stringent site selection requirements and residents’ serious lack of awareness of ageing national conditions, untimely and unsmooth communication with residents, the NIMBY attitude has been accumulated [9].

Although most of the public recognizes the contribution of the CACSC to society, many community residents have resisted. This study suggests that the establishment of the CACSC is similar to facilities such as power plants, garbage dumps, and chemical plants. The CACSC may have an avoidance effect on disgusting facilities. Most existing studies address the NIMBY attitude of community residents towards the CACSC network through a qualitative analysis of actual cases and their attitudes and perceptions towards the CACSC network. There is still a lack of sufficient empirical research on related topics; this issue requires in-depth research and observation.

Therefore, this study explores the opposition intentions of community residents over the age of 20 to the establishment of a CACSC in the community and related factors. Based on the theory of NIMBY, perceived knowledge (PK), superstitious belief (SB), perceived risk (PR), perceived benefit (PB), and NIMBY attitudes (NA) are the factors that influence community residents’ opposition intention (OI) towards the establishment of the CACSC network. To actively respond to the ageing population, we provide relevant suggestions to the government and CACSC companies and adopt policies or related measures to improve the harmony between community residents and CACSCs, which is conducive to building a model community that cares for the elderly.

### Conceptual model

**Fig. 2:**
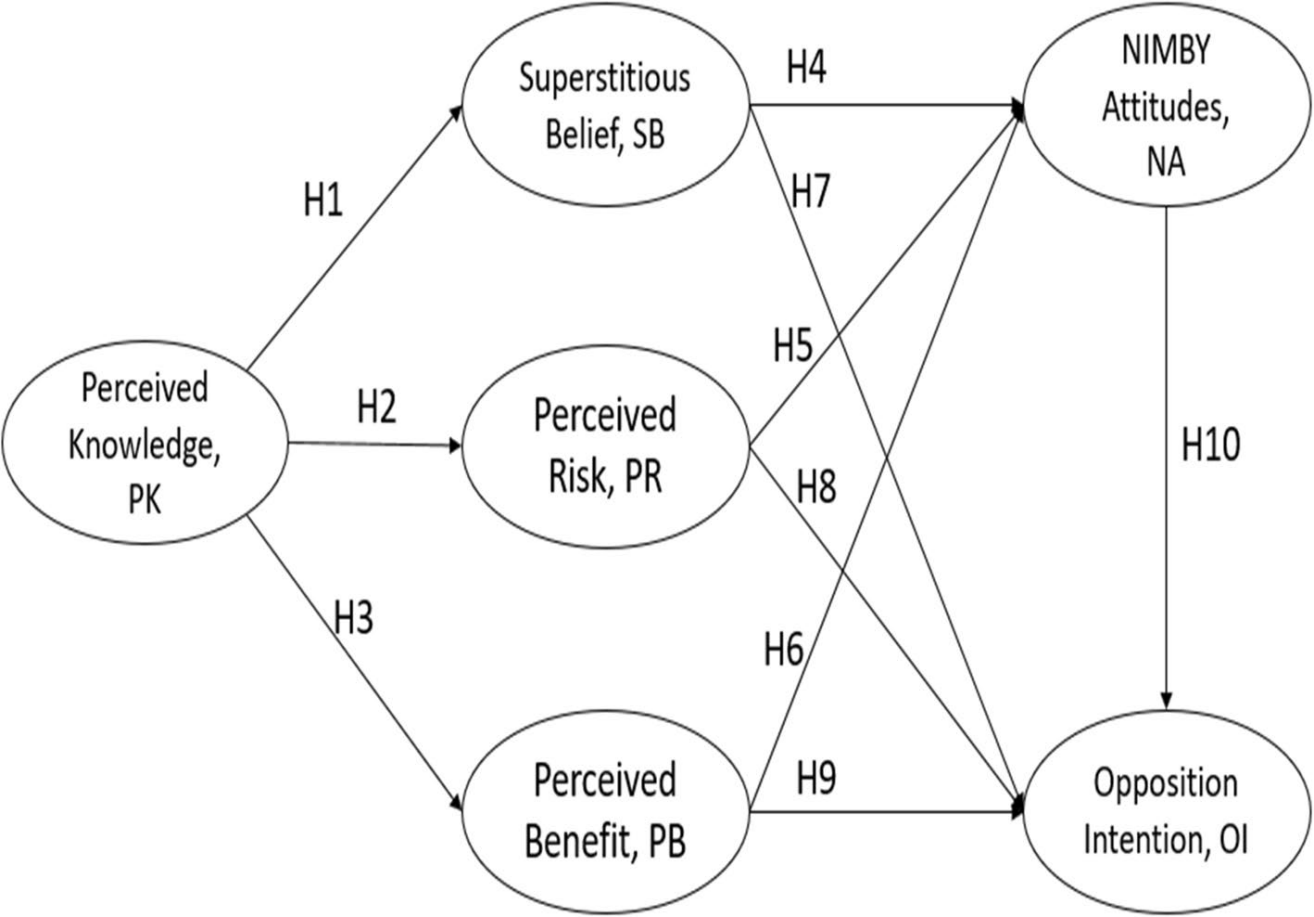
Conceptual model

The conceptual model is proposed as shown in Fig. 2, includes both direct and indirect relationships. Specifically, this study proposes that PK is directly related to SB, PR and PB, SB, PR and PB are related to NA and OI. PK is indirectly related to NA and OI. Therefore, Fig. 2 illustrates how NA influences OI. In the following sections, we discuss each of the factors and hypothesize linkages between them.

### Perceived knowledge

History has indicated that knowledge is power and that the development of human society cannot be separated from the advancement of knowledge [10]. Knowledge has an important role in explaining individual attitudes and behaviours and affects individual decision-making [11]. In existing empirical research, knowledge is often divided into two structures: objective knowledge and subjective knowledge [12]. Objective knowledge is accurate information about certain things stored in long-term memory, and subjective knowledge refers to people’s views about a certain things [13].

Studies have shown that objective knowledge assessment and subjective knowledge assessment are often positively correlated, and subjective knowledge is better measured than objective knowledge [14]. Therefore, this study uses subjective knowledge to evaluate, that is, to measure the understanding of community residents about the trend of social ageing and the relevant knowledge of the CACSC network.

### Superstitious belief

In many cultures and societies, superstition is considered an influencing factor on people’s attitudes and behaviours. The definition of SB is based on unknown or mysterious power rather than scientific knowledge, that is, specific behaviours can produce specific results [15]. Some scholars suggest that superstition is not correlated with people’s socioeconomic or educational status [16]. Studies have confirmed that superstition affects consumer attitudes and behaviours [17]. In addition to consumer behaviours, superstitions are also employed to study the influence on other attitudes and behaviours. For example, Lu et al. [18] investigated the influence of SB on the intention of vaccination through PB and knowledge of influenza. Tarrant et al. [19] found that SB affects South Africans’ attitudes towards frogs and thus affect the protection of amphibians.

This study suggests that the attitudes and opinions of community residents towards the CACSC will be affected by the degree of SB of the residents themselves, and the SB here refers to the residents’ fear of encountering ghosts or that deaths of the elderly in the CACSC would affect feng shui and personal luck. Studies have shown that the stronger the PK, the weaker the degree of SB [19], that is, the stronger the community residents’ PK of social ageing trends and CACSCs is, the weaker their superstitious concept of the CACSC. Therefore, the following hypothetical statement is presented:

- H1: Community residents’ PK of social ageing trends and CACSCs has a negative impact on SB.

### Perceived risk

In the study of individual attitudes and behaviours, PK is an important psychological variable [20]. PK refers to an individual’s perception of the uncertainty and possible negative consequences of a specific event or behaviour [21]. Negative consequences include adverse effects on personal health and society [22]. A study by Weyman et al. [23] found that the public’s PK is affected by the degree of trust in an organization. PK is also affected by the distance between people and the perceived hazard. The closer the distance is, the higher the PK [24].

Research on the PK of public facilities focuses on energy facilities, such as nuclear power plants (especially after the accident at the Fukushima nuclear power plant in Japan) and geothermal energy [25–27]. In this study, PK mainly refers to the economic, social, environmental, and physical and mental health concerns of local community residents about the construction and operation of CACSCs. Studies have shown that PK will have a significant negative impact on PK [25, 28], that is, the stronger the PK of community residents on social ageing trends and CACSCs is, the weaker their PK. Therefore, the following hypothetical statement is presented:

- H2: Community residents’ PK of social ageing trends and CACSCs has a significant negative impact on PK.

### Perceived benefit

The public’s acceptance attitude is affected by not only PK but also PB [26], and the stronger the PB is, the lower the PK [29]. PB refers to the perceived possibility that a suggested action will produce a positive result [30]. Moreover, PB is a cognitive-emotional structure that can have a positive impact on individual behaviour [31].

PB in this study refers to the extent to which local residents believe that the entire society, families and individuals will benefit from the construction and operation of CACSCs. PB would then be positively affected by public knowledge. The stronger the public’s PK is, the stronger the PB [26]. Therefore, the following hypothetical statement is presented:

- H3: Community residents’ PK of social ageing trends and CACSCs has a significant positive impact on PB.

### NIMBY attitude

In the 1970s, in the study of urban and environmental public policy, researchers discovered which facilities residents believed were necessary to build despite their concern that facilities built near their homes would have negative impacts on their own health, environment, and property value: the NIMBY attitude. This attitude inspired a people’s disgust complex and even led to strong resistance behaviours [32]. Therefore, residents’ opposition would lead to tensions between the community and the facilities and impacted the interests of customers who used these facilities and desired social harmony [33].

Research on the NIMBY theory focuses on the public’s attitudes and opposition to the construction of avoidance facilities, such as nuclear power plants, garbage dumps, chemical plants, and prisons [34–36]. Different from the abovementioned avoidance facilities, the negative impacts of CACSCs on residents’ health and the environment is not obvious, and the impact of such facilities on residents is more psychological in nature. For example, Dear and Taylor [37] pointed out that the NA of community residents about the construction of community mental health facilities was influenced by the negative attitudes of community residents towards people with mental health problems. Nevertheless, in practical terms, studies have also confirmed that in China, death-related facilities such as funeral homes can reduce the price of surrounding houses by 3.2% [38]. Existing studies have confirmed that NA are positively affected by superstition and PK and negatively affected by PB. Therefore, the following three hypotheses are proposed:

- H4: Community residents’ SB has a significant positive impact on NA towards the construction of CACSCs.
- H5: Community residents’ PK has a significant positive impact on NA towards the construction of CACSCs.
- H6: Community residents’ PB has a significant negative impact on NA towards the construction of CACSCs.

### Opposition intention

Behavioural intention and actual behaviour are indicators that are often employed when measuring people’s behavioural responses [24]. Behavioural intentions will affect actual behaviour and can accurately predict behaviour [39]. In addition, since actual behaviour is difficult to measure, behavioural intention is often measured in research [40].

The OI in this study refers to the negative behaviour intentions of community residents to resist the construction of the CACSC due to the NIMBY attitude. In research on the influencing factors of OI, SB is considered a significantly negative influence. For example, Lu et al.’s [18] study on influenza vaccination of Singapore residents found that the stronger their belief in superstition, the greater the likelihood that a person would not receive the flu vaccine. Moreover, when predicting people’s behavioural intentions in risky situations, PK and PB are often regarded as important factors. For example, Choi et al. [41] examined consumers’ attitudes and behavioural intentions towards street food and found that PK has a negative impact on consumers’ behavioural intentions towards street food. He [42] investigated 237 users of the DiDi platform in mainland China and found that PB had a positive impact on the willingness to continue sharing, while PK had a negative impact. A large number of studies have shown that attitudes have a significant positive impact on behavioural intentions. Therefore, the following four hypotheses are proposed:

- H7: Community residents’ SB has a significant positive impact on the OI towards the construction of CACSCs.
- H8: Community residents’ PK has a significant positive impact on the OI towards the construction of CACSCs.
- H9: Community residents’ PB has a significant negative impact on the OI towards the construction of CACSCs.
- H10: Community residents’ NIMBY attitude has a significant positive impact on the OI towards the construction of CACSCs.

## Methods

### Data collection

**Fig. 3:**
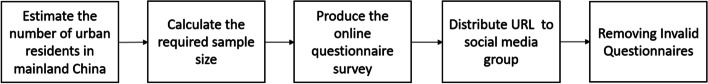
Flow diagram of the data collection procedure

The subjects of this study are residents of urban communities over the age of 20 in mainland China. According to the seventh census of the National Bureau of Statistics of China, the permanent population of urban residents in mainland China was 900.99 million at the end of 2020. Using the sample size calculator on the Survey System website, at the 95% Confidence Interval (CI), the minimum required number of samples was 384. Compared with the offline paper questionnaire survey, the online questionnaire survey method has the advantages of cost savings, time flexibility, high efficiency, no geographical restrictions, no missing values, and accurate data entry [43, 44]. Therefore, this study adopts an online questionnaire survey method to collect sample data. The Chinese online survey platform Questionnaire Star (https://www.wjx.cn/) was selected to distribute the online questionnaires. In this study, all questions were uploaded to the website to generate an online questionnaire. By sending the uniform resource locator (URL) link to social media groups in different regions through WeChat, the researcher efficiently collected samples. Since responding to all questions in the online questionnaire is mandatory, the questionnaires could only be submitted after all questions were answered. Therefore, there were no missing values in the collected data. This study collected a total of 546 sample data points, excluding data from respondents under the age of 20, sample data with the same answer, and sample data with excessively short response times. A total of 509 valid questionnaires were collected. The effective questionnaire recovery rate was 93.22%. Figure 3 is a flow diagram of the data collection procedure.

### Ethics

This study was conducted according to the guidelines of the Declaration of Helsinki. Ethical approval of this study was obtained from the Medical Ethics Committee of the Kunming University of Science and Technology [KMUST-MEC-086] before start of the survey. Written informed consent was obtained from all participants prior to filling in the survey with assurance of confidentiality of the data.

### Measurement instrument

This research questionnaire is divided into two parts. The first part consists of the demographic variables of the interviewees, including gender, age, education level, residential area, and current living conditions. The second part comprises the structure of the conceptual model (Fig. 2), which contains six latent variables: PK, SB, PK, PB, NA, and OI. The measurement questions are modified by previous research based on the specific scenarios of this research. These measurement questions are all written in English by the researchers except for PK. To ensure the accuracy of the translation, after the questions were translated into Chinese with translation software, an English major graduate student was invited to help with proofreading the questionnaires, and two scholars working in community governance and ageing research were invited to assess the validity of the questionnaire content. All measurement items are measured using a seven-point Likert scale, and the measurement of NA is based on “no objection to construction at all” (1) to “very opposed to construction” (7). Other topics are based on ratings from “strongly disagree” (1) to “strongly agree” (7) to perform the measurement.

The measurements of latent variables were designed to follow previous studies. For example, SB measurement was generally applied to the Paranormal Belief Scale evaluated by Tobacyk and Milford [45]. Žeželj et al. [16] then extended this research on the basis of this scale. This study modified the scale according to the research theme and traditional Chinese concepts, such as “Building a CACSC will destroy feng shui” and “A CACSC is a relatively unlucky place”. Moreover, the measurement of PK was modified from the PK scale of adjacent avoidance facilities investigated by Chinese scholars [46]. This study provided six measurement topics, such as “I am worried that the construction and operation of a CACSC in the community where I live will reduce the housing price of the community” and “I am worried that the construction and operation of a CACSC will increase the flow of people and reduce the safety of the community”. The PB Scale refers to the scale of Ok et al. [47]. From the perspectives of social and personal benefits, five measurement questions were designed, for example, “I think the CACSC is beneficial to society”, and “I think the CACSC is beneficial to the elderly”. In addition, based on the measurement of perceptual knowledge in the study of Zhu et al. [25], the five measurement questions of perceptual knowledge obtained in this study are modified, such as “I understand the trend and severity of social ageing” and “I know the country’s policies on actively responding to ageing”. Furthermore, NA and OI were referenced by Zeng et al. [34]. There are four measurement questions of NA: “I oppose the construction of a CACSC as it may harm personal interests” and “I oppose the construction of a CACSC as it may harm personal health (including mental health and physical health)”. The OI measurement includes five items, such as “I will publicly express my opposition to the construction of a CACSC” and “I will sign the petition against the construction of a CACSC”.

### Data analysis

The data analyses of this study were processed in three sections. First, we selected IBM SPSS Statistics 25.0 as the software to perform descriptive statistical analysis, such as the frequency distribution calculation of demographic variables and the mean value and standard deviation of the items of each construct. In addition, T-tests and ANOVA were performed to conduct a differential analysis of the project averages of different groups. Second, using IBM SPSS Amos 24.0 statistical software for measurement model verification, the structural equation modelling method of covariance-based structural equation modelling (CB-SEM) verifies the reliability and validity of all variables. Third, we analyse the structural equation model by the fit of the model and verify the assumptions among the variables.

## Results

### Descriptive statistical analysis

#### Frequency distribution

In this study, demographic data such as gender, age, education, living area, property, living with elderly individuals, and CACSCs in the community were investigated. Regarding gender, females accounted for the largest number (320, 62.87%). Regarding age, 41–60 years old represented 217 samples or 42.63%. For education, most of the participants were undergraduate college students (286, 56.19%). Regarding living area, most of the sample data (167, 32.81%) was collected in the southwest area. For property, most of the participants were owners of houses (448, 88.02%). There were 267 participants living with the elderly (52.46%). A total of 85.85% of the participants’ communities lacked CACSCs, as shown in Table 1.

**Table 1:** General Characteristics of the study sample (*N* = 509)

Characteristics	Categories	n	Percent(%)
Gender	Male	189	37.13
	Female	320	62.87
Age	21–40 years old	212	41.65
	41–60 years old	217	42.63
	61 years or above	80	15.72
Education	Junior school or below	52	10.22
	High school/vocational school	80	15.72
	College/university	286	56.19
	Master or above	91	17.88
Living area	Northeast China	78	15.32
	Northwest China	22	4.32
	North China	116	22.79
	Central China	41	8.06
	East China	45	8.84
	South China	40	7.86
	Southwest China	167	32.81
Property	Purchase	448	88.02
	Lease	61	11.98
Living with elderly	Yes	267	52.46
	No	242	47.54
CACSC in community	Yes	72	14.15
	No	437	85.85

#### Item statistical analysis

The mean values of all items ranged from 1.66 to 6.14, and the standard deviations were between 1.43 and 2.20, showing the consistency in the response to each question by the participants. Moreover, the skewness ranged from −2.08 to 2.44, and the kurtosis values ranged from − 1.29 to 5.34, which are qualified with the results that the absolute value of skewness is less than 2 and the absolute value of kurtosis is less than 7 [48]. Thus, the data are normally distributed, as shown in Table 2.

**Table 2:** Descriptive analysis table

Variable	Item	Mean	SD	Kurtosis	Skewness
PK	PK1	5.85	1.73	1.37	−1.54
	PK2	5.30	1.82	−.26	−.84
	PK3	5.64	1.72	.69	−1.25
	PK4	4.59	1.99	−1.04	−.29
	PK5	5.28	1.80	−.30	−.81
SB	SB1	1.85	1.67	3.19	2.06
	SB2	1.66	1.43	5.34	2.44
	SB3	2.04	1.68	1.57	1.60
	SB4	2.44	1.88	.09	1.13
PR	PR1	2.21	1.77	1.00	1.42
	PR2	2.67	1.96	−.29	.95
	PR3	3.52	2.20	−1.29	.30
	PR4	3.01	2.03	−.81	.66
	PR5	2.48	1.77	.20	1.07
	PR6	2.53	1.84	.16	1.09
PB	PB1	5.98	1.71	2.19	−1.79
	PB2	6.14	1.57	3.48	−2.08
	PB3	6.07	1.62	2.93	−1.96
	PB4	6.00	1.57	2.26	−1.72
NA	NA1	2.03	1.58	2.13	1.70
	NA2	2.03	1.53	1.89	1.61
	NA3	1.98	1.45	2.11	1.64
	NA4	2.04	1.48	2.07	1.61
OI	OI1	2.01	1.71	2.56	1.9
	OI2	2.04	1.79	1.84	1.75
	OI3 OI4	1.98 2.00	1.63 1.69	2.28	1.78
				2.04	1.76
	OI5	1.93	1.74	2.45	1.92

*SD* standard deviation, *PK* perceived knowledge, *SB* superstitious belief, *PR* perceived risk, *PB* perceived benefit, *NA* NIMBY attitudes, *OI* opposition intention

#### T-test

For the PK, PB, and OI variables, the mean scores of people who own their houses (purchase) are greater than those who lease their houses (lease). On the other hand, for the SB, PR, and NA variables, the mean scores of the owners (purchase) are less than those of the tenants (lease), except for the NA, where the scores of the owners are significantly higher than those of the tenants (*P*<.05). The other constructs have no significant difference between the owners and the tenants (Table 3).

**Table 3:** T-test by property

Variable	Property	n	Mean	SD	T-test	df	***P-***value Sig.
PK	Purchase	448	5.34	1.56	.20	507.00	.842
	Lease	61	5.30	1.41			
SB	Purchase	448	1.98	1.34	−.51	507.00	.613
	Lease	61	2.08	1.36			
PR	Purchase	448	2.71	1.54	−1.08	507.00	.280
	Lease	61	2.93	1.53			
PB	Purchase	448	6.05	1.51	.18	507.00	.856
	Lease	61	6.02	1.43			
NA	Purchase	448	1.96	1.32	−2.41	507.00	.016
	Lease	61	2.41	1.69			
OI	Purchase	448	1.99	1.46	.01	507.00	.995
	Lease	61	1.99	1.34			

*SD* standard deviation, *df* degree of freedom, *Sig.* significant, *PK* perceived knowledge, *SB* superstitious belief, *PR* perceived risk, *PB* perceived benefit, *NA* NIMBY attitudes, *OI* opposition intention

For the SB, PR, NA, and OI variables, the mean scores of the participants who lived in the community with CACSCs were greater than those who lived in communities without CACSCs. For the PB and PK variables, the mean scores of participants who lived in communities with CACSCs were less than those of participants who lived in communities without CACSCs. Only the SB and OI variables for those who lived in communities CACSCs were significantly higher than those who lived in communities without CACSCs (*P*<.05), as shown in Table 4.

**Table 4:** T-test by centre

Variable	Centre	n	Mean	SD	T-test	df	***P-***value Sig.
PK	Yes	72	5.21	1.82	−.73	507.00	.463
	No	437	5.35	1.49			
SB	Yes	72	2.40	1.81	2.74	507.00	.006
	No	437	1.93	1.24			
PR	Yes	72	2.98	1.78	1.49	507.00	.138
	No	437	2.69	1.50			
PB	Yes	72	5.80	1.78	−1.55	507.00	.121
	No	437	6.09	1.45			
NA	Yes	72	2.13	1.52	.73	507.00	.463
	No	437	2.00	1.35			
OI	Yes	72	2.54	1.87	3.50	507.00	.001
	No	437	1.90	1.34			

*SD* standard deviation, *df* degree of freedom, *Sig.* significant, *PK* perceived knowledge, *SB* superstitious belief, *PR* perceived risk, *PB* perceived benefit, *NA* NIMBY attitudes, *OI* opposition intention

#### ANOVA

By analysing different age levels for the variable SB, the F value of the SB is significant (F = 6.24, *P* = .002<.05), indicating that at least one pair of the means is different. The post hoc comparison using the Scheffe method reveals that the elderly subjects above 61 years old have significantly higher SB degrees than those aged 21–40 years old (Table 5).

**Table 5:** Analysis of variance by age

Variable	Age	n	Mean	SD	F-text	***P***-value Sig.	Scheffe
PK	21–40 years old	212	5.05	1.56	8.50	.000	2>1, 3>1
	41–60 years old	217	5.42	1.57			
	61 years or older	80	5.84	1.24			
SB	21–40 years old	212	1.78	1.06	6.24	.002	3>1
	41–60 years old	217	2.07	1.51			
	61 years or older	80	2.37	1.46			
PR	21–40 years old	212	2.59	1.35	2.61	.074	
	41–60 years old	217	2.77	1.62			
	61 years or older	80	3.04	1.73			
PB	21–40 years old	212	5.94	1.62	1.19	.306	
	41–60 years old	217	6.10	1.41			
	61 years or older	80	6.21	1.39			
NA	21–40 years old	212	1.83	1.15	3.52	.030	No difference between groups
	41–60 years old	217	2.15	1.51			
	61 years or older	80	2.17	1.50			
OI	21–40 years old	212	1.44	.68	30.20	.000	2>1, 3>1
	41–60 years old	217	2.41	1.67			
	61 years or older	80	2.33	1.75			

*SD* standard deviation, *Sig.* significant, *PK* perceived knowledge, *SB* superstitious belief, *PR* perceived risk, *PB* perceived benefit, *NA* NIMBY attitudes, *OI* opposition intention

The F values of PR (F = 2.61, *P* = .074>.05) and PB (F = 1.19, *P* = .306>.05) were not significant. There was no mean difference in different age levels for the PR and PB variables.

The F value of the PK was significant (F = 8.50, *P* = .000<.05), indicating that at least one pair of the means at different age levels was different. The post hoc comparison using the Scheffe method revealed that 41–60-year-old subjects had significantly higher PK degrees than 21–40-year-old subjects. The elderly subjects above 61 years old had significantly higher PK degrees than 21–40 years old.

The F value of the NA was significant (F = 3.52, *P* = .030<.05), indicating that at least one pair of the means at different age levels in the NA variable was different. After the Scheffe post hoc comparison, there was no difference between the groups. The F value of the OI was significant (F = 30.20, *P* = .000<.05), indicating that at least one pair of means at different age levels was different. The post hoc comparison using the Scheffe method reveals that the subjects aged 41–60 years old have significantly higher OI degrees than those aged 21–40 years old. The elderly subjects above 61 years old had significantly higher degrees of OI than those aged 21–40 years old.

### Measurement model verification

#### Convergent validity

This study assessed the measurement and structural model adopting the two-step approach of structural equation modelling (SEM) proposed by Anderson and Gerbing [49]. The first step using confirmatory factor analysis (CFA) examined the construct reliability and validity of the measurement model. The second step tested the path effects and their significance of the structural model. By using maximum likelihood estimation (MLE) in terms of factor loadings, reliability of measurement, convergent validity, and discriminant validity, the measurement model was assessed.

The standardized factor loading should be .7 or above, and a factor above .6 was acceptable. All standardized factor loadings of items range from .677 to .928, falling into a reasonable range. This result demonstrates that all questions have convergent validity. All the composite reliability of the constructs ranging from .826 to .946 exceed the .7 recommended by Nunnally and Bernstein [50], indicating that all constructs have internal consistency. All average variance extracted (AVE) ranging from .545 to .815 exceed .5 as indicated by Hair et al. [51] and Fornell and Larcker [52], showing that all constructs have adequate convergent validity (Table 6).

**Table 6:** Results for the measurement model

Construct	Item	Significance of Estimated Parameters				Item Reliability		Construct Reliability	Convergence validity
		Unstd.	S.E.	Unstd./S.E.	***P***-value	Std.	SMC	CR	AVE
PK	PK1	1.000				.768	.590	.907	.661
	PK2	1.183	.058	20.443	.000	.863	.745		
	PK3	1.130	.053	21.213	.000	.871	.759		
	PK4	1.133	.066	17.060	.000	.755	.570		
	PK5	1.085	.058	18.598	.000	.801	.642		
SB	SB1	1.000				.680	.462	.826	.545
	SB2	.999	.063	15.812	.000	.795	.632		
	SB3	1.167	.089	13.089	.000	.791	.626		
	SB4	1.117	.096	11.607	.000	.677	.458		
PR	PR1	1.000				.710	.504	.887	.567
	PR2	1.225	.074	16.654	.000	.786	.618		
	PR3	1.245	.084	14.860	.000	.711	.506		
	PR4	1.332	.079	16.882	.000	.822	.676		
	PR5	1.075	.067	16.003	.000	.763	.582		
	PR6	1.054	.070	15.113	.000	.720	.518		
PB	PB1	1.000				.872	.760	.946	.815
	PB2	.976	.031	31.542	.000	.928	.861		
	PB3	1.005	.033	30.712	.000	.924	.854		
	PB4	.930	.033	27.961	.000	.885	.783		
NA	NA1	1.000				.850	.722	.933	.776
	NA2	1.053	.036	29.186	.000	.924	.854		
	NA3	.996	.036	27.650	.000	.919	.845		
	NA4	.911	.040	22.982	.000	.826	.682		
OI	OI1	1.000				.730	.533	.900	.646
	OI2	1.094	.063	17.378	.000	.765	.585		
	OI3	1.181	.059	20.107	.000	.902	.814		
	OI4	1.235	.061	20.187	.000	.912	.832		
	OI5	.955	.063	15.139	.000	.684	.468		

*Unstd.* unstandardized factor loading, *S.E.* standard error, *Std.* standardized factor loading, *SMC* squared multiple correlations, *CR* composite reliability, *AVE* average variance extracted, *PK* perceived knowledge, *SB* superstitious belief, *PR* perceived risk, *PB* perceived benefit, *NA* NIMBY attitudes, *OI* opposition intention

#### Discriminant validity

For discriminant validity, the square root of the AVE of a given construct was compared with the correlations between this construct and the other constructs [52]. If the square root of the AVE of a construct is greater than the off-diagonal elements in the corresponding rows and columns, then the indicators are more closely related to the construct than the other constructs. The bold numbers in the diagonal direction represent the square roots of the AVEs, which are greater than the off-diagonal numbers. Therefore, the discriminant validity appears to be satisfactory for all constructs (Table 7).

**Table 7:** Discriminant validity for the measurement model

	AVE	PK	SB	PR	PB	NA	OI
PK	.661	**.813**					
SB	.545	−.171	**.738**				
PR	.567	−.124	.021	**.753**			
PB	.815	.670	−.114	−.083	**.903**		
NA	.776	−.185	.291	.409	−.198	**.881**	
OI	.646	−.133	.446	.232	−.115	.414	**.804**

*AVE* average variance extracted*, PK* perceived knowledge*, SB* superstitious belief*, PR* perceived risk*, PB* perceived benefit*, NA* NIMBY attitudes*, OI* opposition intention

### Structural equation model

#### Model fit

In the SEM analysis, a good model fit demonstrates that the covariance matrix generated by the sample is consistent with the expected covariance matrix produced by the investigated model. This study uses several model fit indicators that have been examined and that fall within a reasonable range of criteria, as shown in Table 8.

**Table 8:** Model fit of research model

Model fit	Criteria	Model fit of research model
χ^2^	The small the better	924.174
df	The large the better	340.000
Normed Chi-square (χ^2^/df)	1 < χ^2^/df < 3	2.718
RMSEA	<.08	.058
TLI (NNFI)	>.9	.907
CFI	>.9	.917
GFI	>.9	.916
AGFI	>.9	.907
Scaling correction factor	> 1	1.461

*χ*^*2*^ chi-square, *df* degree of freedom, *RMSEA* root mean square error of approximation, *TLI (NNFI)* Tucker-Lewis index (non-normed fit index), *CF*I comparative fit index, *GFI* goodness-of-fit index, *AGFI* adjusted goodness-of-fit index

#### Path analysis

**Fig. 4:**
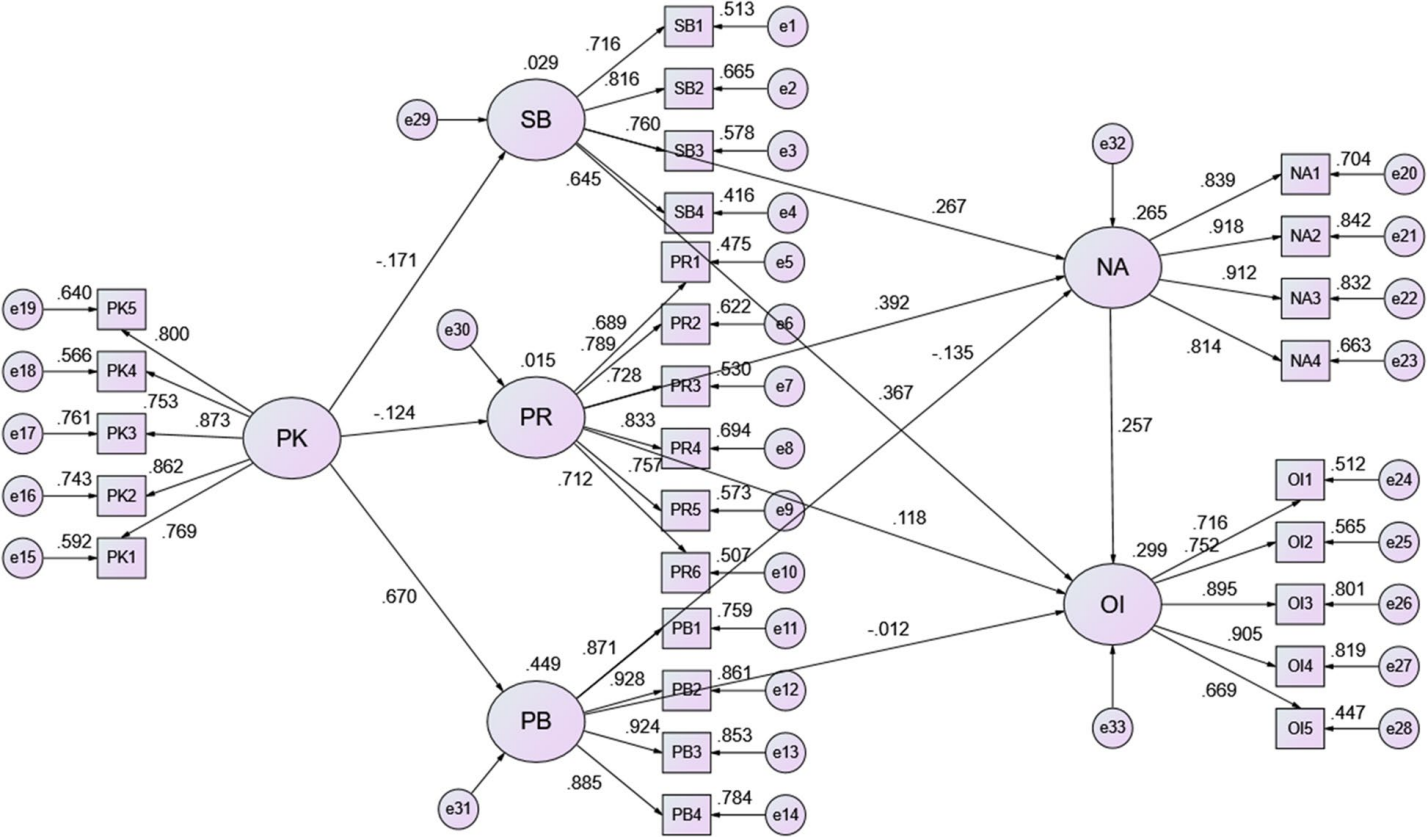
Path diagram for the hypothetical model

PK can significantly influence SB (b = −.154, *P* < .05), PK (b = −.113, *P* < .05), and PB (b = .752, *P* < .001), which suggests that H1, H2 and H3 are supported. The explainable variations were .029, .015, and .449. SB (b = .286, *P* < .001), PK (b = .413, *P* < .001), and PB (b = −.116, *P* < .001) can significantly and positively affect NA, which meant that H4, H5, and H6 were supported. The explainable variation was .265. SB (b = .368, *P* < .001), PK (b = .116, *P* < .05), and NIMBY attitude (b = .240, *P* < .001) can significantly and positively affect OI, which meant that H7, H8, and H10 were supported. Moreover, H9 was not supported since the *P* value is greater than .05, which means that PB has no significant impact on OI. The explainable variation was .299 (Table 9). Figure 4 shows the regression coefficients of the structural equation model.

**Table 9:** Path analysis

DV	IV	Unstd.	S.E.	Unstd./S.E.	***P***-value	Std.	R^**2**^
SB	PK	−.154	.046	−3.342	.001	−.171	.029
PR	PK	−.113	.045	−2.508	.012	−.124	.015
PB	PK	.752	.052	14.406	.000	.670	.449
NA	SB	.286	.060	4.793	.000	.267	.265
	PR	.413	.058	7.101	.000	.392	
	PB	−.116	.037	−3.181	.001	−.135	
OI	SB	.368	.060	6.084	.000	.367	.299
	PR	.116	.054	2.137	.033	.118	
	PB	−.010	.034	−.283	.777	−.012	
	NA	.240	.048	4.951	.000	.257	

*DV* dependent variable, *IV* independent variable, *Unstd.* unstandardized regression coefficients, *S.E.* standard error, *Std.* standardized regression coefficients, *R*^*2*^ explainable variations. *SB* superstitious belief, *PR* perceived risk, *PB* perceived benefit, *NA* NIMBY attitudes, *OI* opposition intention, *PK* perceived knowledge

## Discussion

### Theoretical implications

Based on the theory of NIMBY, this research mainly explores community residents’ opposition to the establishment of CACSCs and explores its influencing factors. The research framework, related hypotheses and integrating factors such as PK, SB, PB, and PK dimensions were proposed. After collecting data through questionnaire surveys, the model was tested using structural equation models, and the hypotheses were verified.

#### Factors influencing OI of the CACSC

This study verified the hypotheses and identified the following four key points.

SB is the main factor influencing the OI of CACSCs. This result is consistent with the research of Lu et al. [18]. This hypothesis may be attributed to traditional Chinese beliefs that everything related to death is very unlucky And that elderly people would die in CACSCs. Some residents are afraid that the death of elderly people in CACSCs will affect the feng shui of their places of residence. As a result, behaviours such as hanging banners, petitioning the government, and exposing the behaviours to the media will be adopted to oppose the construction of a CACSC.

Unlike previous expectations, the negative impact of PB on OI is not significant. This result is inconsistent with the studies of Cooper et al. [53] and He [42]. However, this finding is very consistent with the phenomenon of NIMBY. Although the residents of the community agree that a CACSC will benefit society and the elderly, the existence of the project will not benefit their own lives. As a result, community residents may have an intention to oppose the construction of the project.

SB and PK have a significant positive impact on NA, while PB has a significant negative impact on NA. Among the relationships, the impact of PK is the highest, with a maximum, non-standardized regression coefficient of .413. These results show that the more SB community residents have, the greater is their perception that the CACSC will threaten their personal lives and the more likely they will develop a NIMBY attitude. These findings are consistent with those of several previous studies [41, 54, 55], emphasizing the important role of PK and PB in NA. In a survey of this research, 85.85% of the survey respondents live in communities that have not built a CACSC. Therefore, economic, social, environmental, or physical and mental health concerns are attributed to ignorance and distrust of CACSCs.

PK has a significant positive impact on PB and a significant negative impact on SB and PK. These results show that when community residents understand the severe situation of social ageing, they will be more likely to understand the benefit of CACSCs to the elderly and society. Therefore, the greater the misunderstanding and distrust that they experience is, the easier to support the construction of CACSCs. This result once again verified the research conclusions of Tarrant et al. [19], Zhu et al. [25], and Wang et al. [26].

#### Investigation results

The scores of the research items show that, on the whole, community residents have an average score of 2.02 for NA, which means they do not oppose construction of CACSCs. Additionally, in a survey of the service content of CACSCs, the most popular services are community canteens, leisure and entertainment, and senior college services (92.4% of cases). The second most popular services are home-based care for the elderly and housekeeping services (72.8% of cases). Following these services, people welcome rehabilitation training for the elderly and medical care services for mild cases (72.1% of cases). Furthermore, among all the reverse questioning aspects, the highest overall average score of 2.73 was obtained for PK. These results show that community residents are concerned about the construction of a CACSC, which they worry will create noise, such as square dance and chorus events. These residents scored the highest, with an average score of 3.52 and a standard deviation of 2.20. Regarding positive questioning, the lowest overall score of 5.33 was obtained for PK. Among the questions, the lowest average score of 4.59 and a standard deviation of 1.99 were obtained, which represents an understanding of the service items and operating modes of the CACSC as construction of the CACSC has not been fully covered. Most communities do not have a CACSC, and residents do not have the opportunity to visit and understand the service items and operating modes of a CACSC. Insufficient knowledge of the service centre will also increase the PK of a CACSC, thereby increasing NA and OI.

#### Differentiation analysis results

The results of the differential analysis show that renters have stronger attitudes towards CACSCs than owners, and there is a statistically significant difference (*P* = .16). Most renters are not the elderly, and the interests of the CACSC do not act on themselves. The respondents who live in a community with a CACSC are more superstitious (*P* = .006), indicating that they are more worried about the death of the elderly living in the CACSC and are more likely to oppose it (*P* = .001). The current lack of interaction between CACSCs and community residents, lack of elderly medical treatment or death will cause psychological shocks to residents and aggravate residents’ superstitions, PK, NA, and intentions of opposition. Moreover, respondents aged 41–60 are more superstitious than those aged 21–40, which may be attributed to the ageing stage, which involves a fear of ageing and death. However, they cannot directly enjoy the convenience brought by CACSCs and tend to be more superstitious, have stronger PK, and have stronger OI.

### Practical implications

In response to the global challenge of population ageing, the United Nations and the World Health Organization advocate “healthy ageing” and “active ageing” [56, 57]. In October 2020, China officially upgraded “actively responding to population ageing” as a national strategy [2]. On June 25, 2021, China’s National Development and Reform Commission, Ministry of Civil Affairs of the People’s Republic of China, and China’s Municipal Health Commission jointly issued the “14th Five-Year Plan” to actively respond to the population ageing project and nursery education construction implementation plan. By 2025, the development directions are to further improve elderly care serving infrastructure conditions, promote the standardization of facilities, improve the level of elderly care services, and gradually build an elderly care service system that is coordinated with home community institutions and that combines medical care and health care [58]. In this elderly care service system, the role of the community is quite critical, and the CACSC can serve as a support system for the elderly at home. The CACSC network is the key strategy of China’s elderly care development in the next few years. Therefore, how to make more community residents accept the CACSC and facilitate its construction and development is very important. According to the results of this study, there are several suggestions to reduce the opposition of community residents to the construction of a CACSC.

#### Strengthen policy advocacy on ageing

Through the investigation of this research, community residents have an insufficient understanding of the dangers of social ageing. Therefore, they cannot deeply understand the significance of the construction of CACSCs, which is not conducive to actively responding to the feasible development of a population ageing project. Therefore, the government can strengthen publicity through community public welfare lectures, distributing publicity materials, and broadcasting public welfare advertisements in public places to improve community residents’ knowledge of social ageing.

#### Clarify the service content of CACSC

In response to the superstitious psychology of community residents regarding the fear of death, strengthening education can be achieved by adjusting the service content of the CACSC. The major goal is to meet the needs of the elderly and improve the quality of life of elderly individuals in the community. The service can focus on providing community canteens, leisure and entertainment, senior colleges, elderly home care, housekeeping services, rehabilitation training, medical care services for mild cases, and daycare services for the elderly. Critical care, hospice care services, and 24-h full-time care services can be provided by professional medical institutions and RACFs. These improvements may reduce SB and avoid the occurrence of some conflicts.

#### Encourage and guide young people to participate in public welfare activities for the elderly

Currently, in China, the proportion of young people who participate in public welfare activities for the elderly is inadequate, leading to insufficient awareness of the lives of the elderly, some superstitions and PK. The lack of PK about the ageing society and the CACSC network is likely to produce NA and OIs. The government and enterprises can jointly organize some young people to participate in public welfare activities for the elderly, such as serving as temporary nursing volunteers, accompanying the elderly in some leisure and entertainment activities, and simulating the life of the elderly through equipment. This engagement would enhance young people’s understanding of the elderly, thereby reducing the intention to oppose the construction of a CACSC.

### Limitations and future research

Although this research has some interesting findings and certain contributions, it still has certain limitations. First, there may be a certain deviation in the relationship among the structures in the survey results of the quantitative research. Follow-up research and experimental research can be employed to further examine the causal relationships among these dimensions. Second, this study only relies on the internet to collect cross-sectional data and has certain requirements for mobile phone operation. The surveyed subjects had a bachelor’s degree or higher degree, accounting for 66.4%. These participants may not fully represent Chinese urban community residents. In future research, offline questionnaires can be supplemented appropriately, and the research results can be further verified in a wider variety of population samples. This research only focuses on exploring the impact of PK, SB, PK, and PB on community residents’ NIMBY attitude and OI to the construction of a CACSC. Other factors, such as discrimination against the elderly, compensation mechanisms, and public participation, could be considered in future research.

## Conclusions

This study confirmed that superstition, the NIMBY attitude, and PK have a significant positive impact on the opposition to the construction of CACSCs, while the negative impact of PB on the OI is not significant. Moreover, PK has a significant positive impact on PB and a significant negative impact on superstition and PK. Reduce the opposition of community residents to the construction of a CACSC should therefore consider these and other influential factors.

## Data Availability

The datasets used and analysed during the current study are available from the corresponding author on reasonable request.

## Abbreviations

CACSC: Community Aged Care Service Centre
NIMBY: Not in My Backyard
PK: Perceived Knowledge
SB: Superstitious Beliefs
PR: Perceived Risk
PB: Perceived Benefit
NA: NIMBY Attitudes
OI: Opposition Intention
SMC: Squared Multiple Correlations
CR: Composite reliability
AVE: Average Variance Extracted
χ^2^: Chi-square
RMSEA: Root Mean Square Error of Approximation
TLI (NNFI): Tucker-Lewis Index (Non Normed Fit Index)
CFI: Comparative Fit Index
GFI: Goodness-of-Fit Index
AGFI: Adjusted Goodness-of-Fit Index
